# Functional analysis of a novel homozygous missense *IVD* gene variant: a case report with dual genetic diagnoses

**DOI:** 10.3389/fped.2025.1494530

**Published:** 2025-02-10

**Authors:** Yuying Zhu, Ke Wu, Hanying Wen

**Affiliations:** ^1^ Prenatal Diagnosis Center, Quzhou Maternal and Child Health Care Hospital, Quzhou, Zhejiang, China; ^2^ Laboratory of Prenatal Diagnosis Center, Quzhou Maternal and Child Health Care Hospital, Quzhou, Zhejiang, China

**Keywords:** Prader–Willi syndrome, isovaleric acidemia, genetic counseling, case report, *IVD* gene

## Abstract

**Background:**

Genomic or exome sequencing is beneficial for identifying more than one pathogenic variation causing blended atypical and/or severe phenotypes. Herein, we are the first to report a 5-year-old boy with the blended phenotypes of infantile hypotonia, severe neurodevelopmental disorder, patent ductus arteriosus, cryptorchidism, obesity, distinctive facial features, and elevated isovaleryl carnitine.

**Methods:**

Trio-based whole-exome sequencing was performed on genomic DNA from peripheral blood samples from the boy and his parents. Functional analysis of the *IVD* variant *in vitro* was performed. Mutant *IVD* gene pcDNA3.1(+)-MUT-3xFlag and control pcDNA3.1(+)-WT-3xFlag mammalian expression vectors were constructed. Both vectors were transformed into HEK293T cells. The assays of relative *IVD* gene mRNA expression, IVD protein expression, and enzymatic activity were used.

**Results:**

Whole-exome sequencing identified a novel homozygous missense variant in the *IVD* gene (NM_002225.5) c.1006T>C (p.Cys336Arg) within a region of homozygosity of 15q11.2-q21.3. Our *in vitro* functional and computer simulation findings revealed that this variant was associated with haploinsufficiency, which resulted in dramatically reducing the formation of IVD protein due to unstable mutant protein and not a lack of mRNA expression.

**Conclusion:**

The boy was diagnosed with the dual genetic disorders of Prader–Willi syndrome and isovaleric acidemia. This case provides a useful reference for genetic counseling for complex and diverse clinical phenotypes. The presence of two or more likely pathogenic or pathogenic variations in an individual with neurodevelopmental phenotypes is not an “exceptional” phenomenon.

## Introduction

Isovaleric acidemia (IVA, OMIM #243500) is an autosomal recessive inborn error of leucine metabolism caused by homozygous or compound heterozygous variants in the *isovaleryl-CoA dehydrogenase* gene (*IVD*; OMIM *607036) on chromosome 15q15 (1). Isovaleryl-CoA dehydrogenase (IVD, EC:1.3.8.4) belongs to the acyl-CoA dehydrogenase family, and it is a mitochondrial matrix enzyme that catalyzes the third step in leucine catabolism. A deficiency of IVD results in the accumulation of isovaleric acid, which is a serious hazard to the central nervous system and leads to IVA. Clinical manifestations of untreated IVA include developmental delay, neuropathological implications, characteristic sweaty foot odor, poor feeding, vomiting, lethargy, and seizures during infancy, childhood, or adolescence (2). Prader–Willi syndrome (PWS, OMIM#176270) is caused by the loss of function of paternally expressed genes in 15q11q13, which could be due to a *de novo* paternal deletion, maternal uniparental disomy (UPD) of the 15q11-q13 region, or the silencing of the paternal expressed imprinting genes (3). Consensus diagnostic clinical criteria for PWS have been developed (4). The main clinical features of PWS are severe hypotonia and feeding difficulties in early infancy, hyperphagia leading to obesity in later infancy or early childhood, intellectual disability, delayed language development, craniofacial anomalies, hypogonadism/hypogenitalism, short stature, and distinctive behavioral problems (5). Although the co-occurrence of multiple (two or more) rare diseases intuitively appears unlikely, it was estimated that approximately 4%–7.5% of patients analyzed by trio-based whole-exome sequencing (trio-WES) had two or more genetic diagnoses (6, 7), with a higher frequency in consanguineous families. Herein, we report a Chinese boy diagnosed with isovaleric acidemia and Prader–Willi syndrome due to a region of homozygosity (ROH), which has not been previously reported. We conducted this study to expand the mutational *IVD* spectrum. It should be noted that more than one genome variation may contribute to complex and diverse clinical phenotypes.

## Materials and methods

### Clinical features

Before her pregnancy, a Chinese woman sought genetic counseling from the Prenatal Diagnosis Center of Quzhou Maternity and Child Health Care Hospital (Zhejiang, China). She reported that her first child (the proband) was a 5-year-old boy born with IVA (detected through newborn screening with elevated isovaleryl carnitine), patent ductus arteriosus (PDA), infantile hypotonia, and cryptorchidism. This boy was naturally conceived with an uneventful normal pregnancy. At the age of 5 years and 6 months, tandem mass spectrometry (MS) revealed he still had an isovaleryl carnitine level of 16.08 μmol/L (C5, normal range: 0.01–0.40 μmol/L). A physical examination showed that he had no obvious dysmorphic features of the hands and feet but he had distinctive facial features (almond-shaped eyes, thin upper lip with downturned corners of the mouth, flat nasal bridge, and low-set ears). At the age of approximately 2 years, his constant craving for food resulted in rapid weight gain. At the age of 5 years and 6 months, an anthropometric examination revealed growth retardation with a height of 102 cm (3rd percentile), overweight with a weight of 24.6 kg (90th percentile), and a body mass index (BMI) of 23.6 (>P_85_) (8). The radiographic atlas method for assessing the skeletal age of the hand and wrist indicated delayed bone age with 3/10 ossification center of the left carpal, ossified metacarpophalangeal bones, and unclosed epiphyseal plate. At the age of 5 years and 10 months, the Griffiths Mental Development Scale-China (GDS-C) (9) showed that this boy had a low gross quotient (GQ) score of 36, which suggested that he was moderately delayed in his cognitive and physical development.

### Whole-exome sequencing

Trio-WES was performed on genomic DNA (gDNA) from peripheral blood samples from the boy and his parents. The xGen™ Exome Research Panel v2 (designed by Integrated DNA Technologies) was used for WES. Quality control (QC) of the DNA library was performed using an Agilent 2100 Bioanalyzer System (Agilent, USA). DNA nanoball (DNB) preps of the clinical samples were sequenced on an ultra-high throughput DNBSEQ-T7 platform (MGI, Shenzhen, China) with a paired-end 150 nt strategy following the manufacturer's protocol.

### Bioinformatic analysis

Sequencing data were analyzed according to our in-house (Chigene Translational Medicine Research Center) procedures. Adapters and low-quality reads were removed, and data quantity and quality were statistically analyzed. The trimmed reads were then mapped to the University of California Santa Cruz (UCSC) GRCh37/hg19 reference genome using the Burrows–Wheeler Aligner (BWA) software. Genome Analysis Toolkit (GATK) software was used for single nucleotide polymorphisms (SNPs) and short (<50 bp) insertion/deletion (indel) calling. The Samtools and Picard software packages were used to generate clean Bam data by removing duplicate data. Variants were annotated for analysis using the single nucleotide polymorphism database (dbSNP), gnomAD exomes database, and Chigene in-house minor allele frequency (MAF) database. Tools for pathogenicity prediction including rare exome variant ensemble learner (REVEL) and phaMissense were used for predicting the possible impact of the variants. DynaMut (10) (a web server) is a well-established normal mode approach used for assessing the stability of a mutant protein. Pathogenicity annotation was conducted according to the American College of Medical Genetics and Genomics (ACMG) guidelines, and the Online Mendelian Inheritance in Man (OMIM), Human Gene Mutation Database (HGMD), and ClinVar databases were used as references for the pathogenicity of each variant.

### Variant classification

As per the ACMG guidelines for interpreting sequence variants, the variants were classified. The classification considered the position of the variant in the human genome, MAF, the pathogenicity prediction of the variant, disease mechanism, clinical phenotypes, literature evidence, and evolutionary conservation.

### Variant verification

Sanger sequencing (BigDye Terminator v3.1 Cycle Sequencing Kit and ABI 3730 Applied Biosystem) was used for further verification of candidate variants. Methylation-specific multiplex ligation-dependent probe amplification (MS-MLPA) (ME028-B1 PWS/AS probe mixture purchased from MRC-Holland) was used for methylation of imprinted regions in the human genome.

### Functional analysis of the *IVD* variant *in vitro*

The mutant *IVD* gene pcDNA3.1(+)-MUT-3xFlag and control pcDNA3.1(+)-WT-3xFlag mammalian expression vectors were constructed and purchased from Wuhan Biorun Biosciences Co., Ltd. HEK293T cells were transfected with expression vectors for two tubes. Then, 6 h after the transfection, we gently removed the lipofectamine-containing medium, replaced the medium with Dulbecco's modified eagle medium (DMEM) containing 5% fetal bovine serum (FBS), and incubated the slide at 37℃ in a 5% CO_2_ incubator until day 2. On day 3, samples were collected and used for a reverse transcription-polymerase chain reaction (RT-PCR) in the *IVD* mRNA expression, protein expression, and enzymatic activity assays.

## Results

### Genetic analysis and confirmation

**Figure 1:**
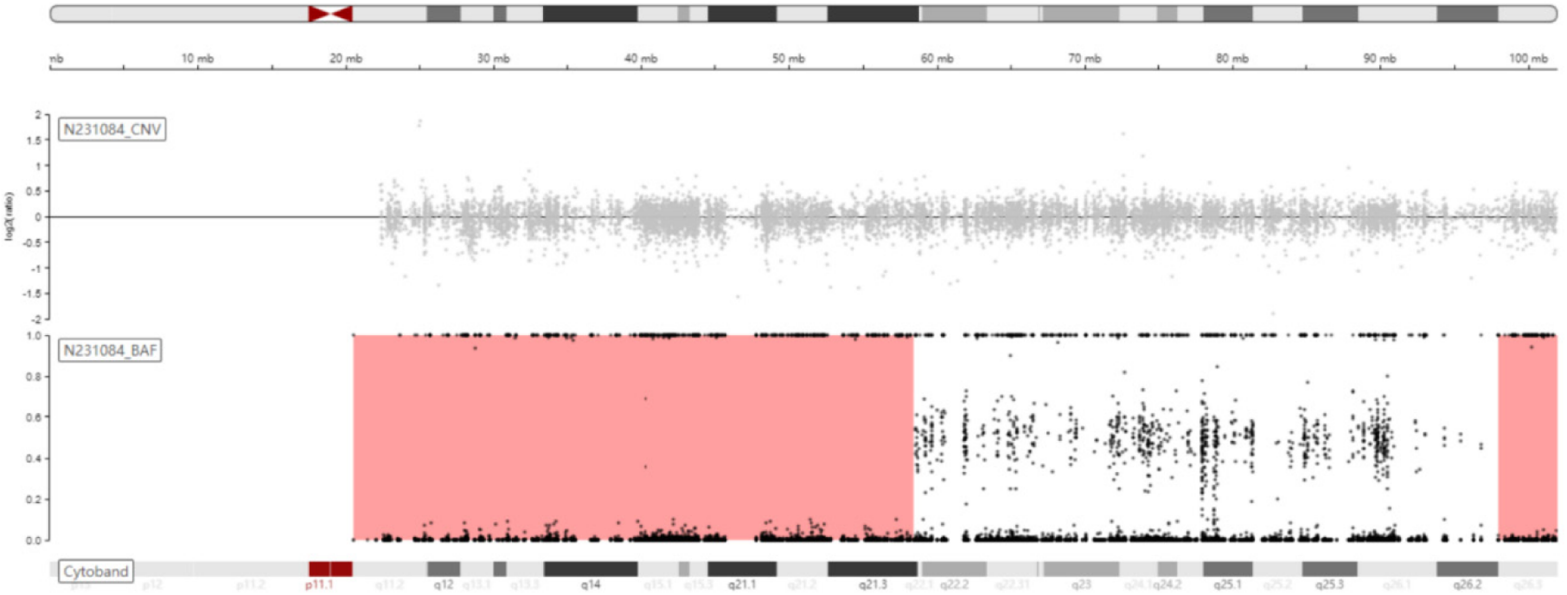
Schematic diagram of B-allele frequency (BAF) on chromosome 15 showing the ROH of 15q11.2-q21.3 (chr15:20534520-58432427, GRCh38).

**Figure 2:**
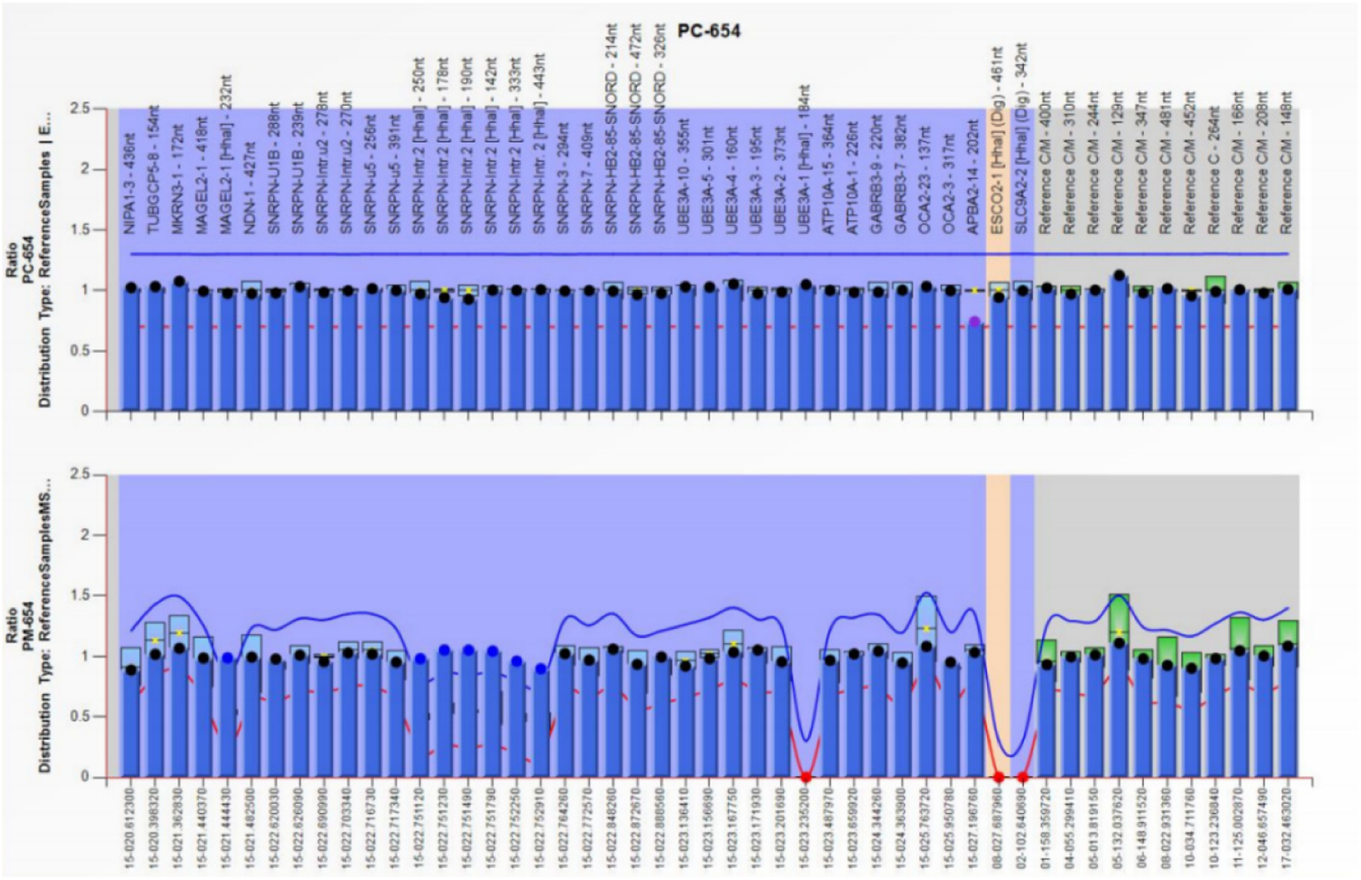
The result of MS-MLPA showing that the copy number of chromosome 15q11.2-q13 was normal but there was abnormal methylation in this region, which suggested this region was maternal UPD.

**Figure 3:**
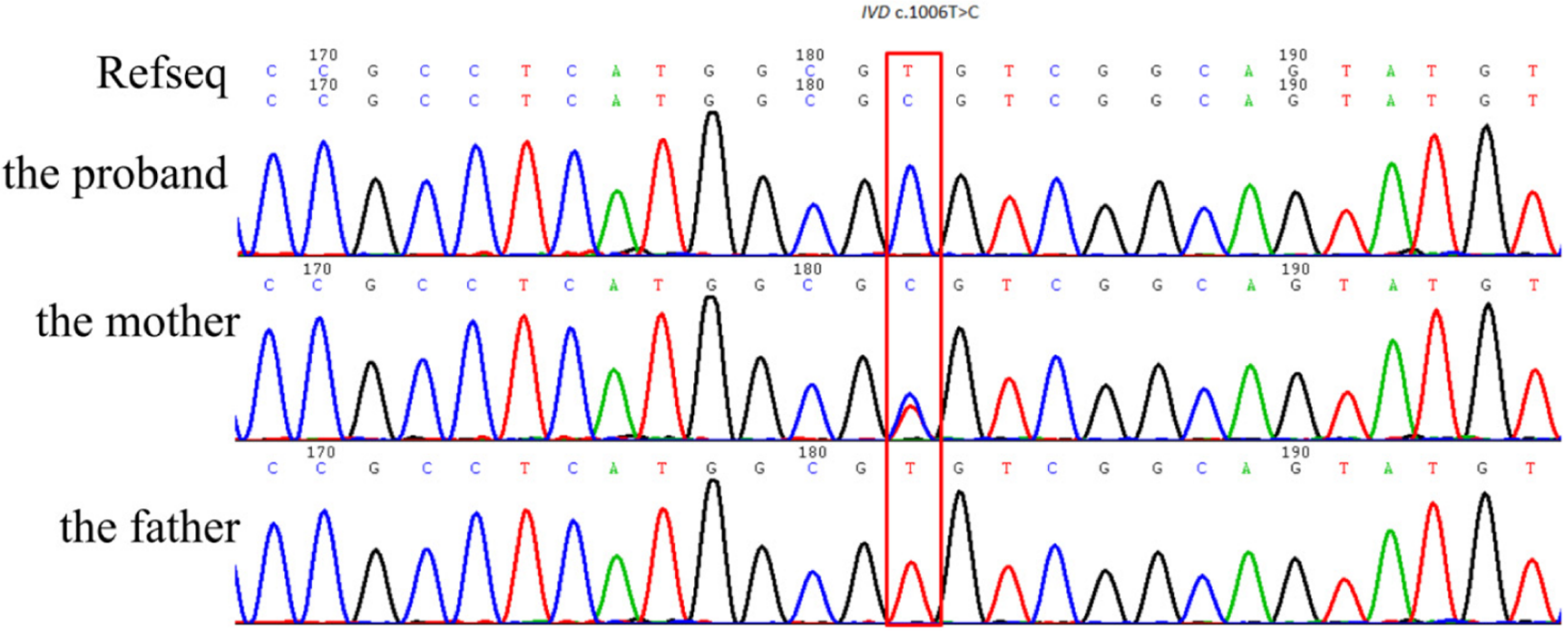
Sanger sequencing confirmed that the homozygous *IVD* gene variant (NM_002225.5) c.1006T>C (p.Cys336Arg) was derived from the boy's heterozygous carrier mother.

Trio-WES identified an ROH in chromosome 15q11.2-q21.3 (GRCh38, chr15:20534520-58432427) (Figure 1) and a novel homozygous *IVD* gene variant (NM_002225.5) c.1006T>C (p.Cys336Arg) caused by an ROH of 15q11.2-q21.3. The ROH of 15q11.2-q21.3 was further verified by MS-MLPA, which revealed maternal UPD (Figure 2). The Sanger sequencing confirmed that the homozygous variant was derived from the boy's heterozygous carrier mother and the boy's father was normal (Figure 3).

The Chinese boy was diagnosed with isovaleric acidemia, which was highly specific for a disease with a single genetic etiology (PP4_Supporting). The allele frequency of homozygous c.1006T>C has not been registered in population databases (1,000 Genomes Project, gnomAD, and dbSNP) or reported in disease databases (ClinVar, Human Gene Mutation Database, and OMIM) (PM2_Supporting). The *in silico* predictive algorithm for this variant showed that the missense variant was moderately deleterious (REVEL score 0.83 > 0.64; AlphaMissense score 0.973 > 0.869) (PP3_Moderate). The DynaMut prediction outcome of the IVD stability was ΔΔG: −0.926 kcal/mol (destabilizing) (PDB accession code: 1IVH; length of mature IVD monomer: 394 amino acids; Cys336 residue mapped to Cys307 in the PDB structure). As per the interpretation guidelines of the American College of Medical Genetics and Genomics (11), this novel variant is classified as a “variant of uncertain significance (VUS)” (PP4; PM2; PP3).

### Functional analysis of the VUS allele

**Figure 4:**
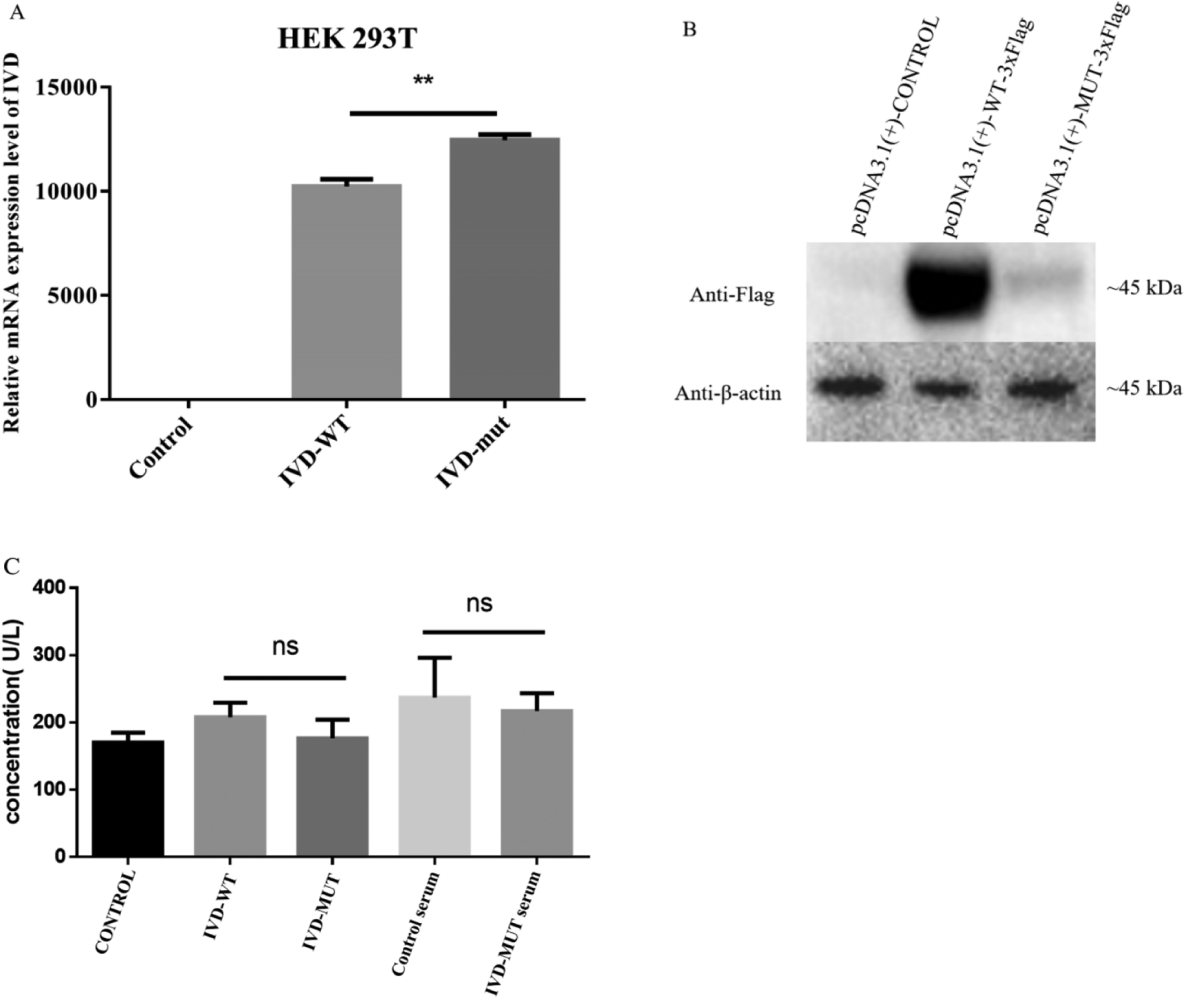
Relative mRNA expression, protein expression, and enzymatic activity of control, wild-type (WT) *IVD,* and mutant *IVD* HEK293T lines. Relative mRNA expression, IVD protein expression, and enzymatic activity assays were all conducted in triplicate. **p < 0.01; ns, no statistical difference. **(A)** Relative mRNA expression of the *IVD* gene variant indicated the level of relative mRNA expression in the *IVD*-WT cell line was significantly lower than in the mutant *IVD* cell line. **(B)** Western blot for IVD confirmed it was significantly reduced in the mutant cell line. Abundant IVD protein was observed with expression of the WT-*IVD* cell line. **(C)** The enzymatic activity assay of IVD (human IVD ELISA Kit) suggested that the enzymatic activity was not significantly different in the mutant HEK293T cell line and patient's serum compared to normal controls, but it was slightly reduced.

To evaluate the effect of this *IVD* VUS allele, our functional study suggested that this variant (NM_002225.5) c.1006T>C (p.Cys336Arg) did not restrict *IVD* mRNA expression from the plasmid (Figure 4A), but led to a lack of IVD protein (Figure 4B). The enzymatic activity assay of IVD (human IVD ELISA Kit) suggested that the enzymatic activity was slightly reduced in the mutant HEK293T cell lines and serum extracted from the patient compared to normal controls (Figure 4C).

## Discussion

The *IVD* gene is located in chromosome 15q15.1. The transcript of *IVD* (NM_002225.5) has 12 exons, a transcript length of 4,350 base pairs, and a translation length of 423 amino acids (12). IVD is a homotetrameric enzyme (172 kDa) with dehydrogenase activity and is composed of four identical mature monomeric peptides (43 kDa). The 43 kDa mature IVD protein is created by posttranslational modification (29 amino acids are removed) of a 45 kDa precursor (in the cytoplasm) initially encoded by the *IVD* gene and is transferred into the mitochondria through the terminal signal peptide. The IVD protein belongs to the acyl-CoA dehydrogenase family (ACAD) and catalyzes the conversion of isovaleryl-CoA(3-methylbutyryl-CoA) into 3-methylcrotonyl-CoA as the third step in the leucine catabolic pathway (13). Ubiquitous expression of IVD is in the thyroid (RPKM 25.0) and liver (RPKM 18.5) (14). IVD contains three domains of approximately equal size: an N-terminal domain, a middle domain, and a C-terminal domain. The genotype–phenotype correlation in patients with IVA has not yet been clarified (15).

These *in vitro* and computer simulation findings were consistent with the previous functional analysis (16) of p.Arg337Gln (c.1010G>A) adjacent to p.Cys336Arg (found by this study). The p.Cys336Arg variant may dramatically reduce the formation of IVD protein due to unstable mutant protein and not a lack of mRNA expression. We speculated that this homozygous missense variant was associated with haploinsufficiency and caused a loss of IVD function, which disrupted the normal function of the enzyme. Furthermore, in the functional study and as per the guidelines of ACMG for interpreting the sequence, this variant was upgraded to “likely pathogenic.”

In neurodevelopmental disorders in the Turkish population, Mitani et al. identified that 28.9% of families (51/176) with more than one pathogenic variant were mostly driven by an ROH (17). In this case, the ROH of 15q11.2-q21.3 included the *IVD* gene variant. The boy presented with two kinds of rare genetic disorders. Thus, it is essential to note that more than one genome variation may contribute to complex and diverse clinical phenotypes.

In conclusion, we are the first to report a boy with dual genetic diagnoses of Prader–Willi syndrome and isovaleric acidemia attributed to a segmental isodisomy. The identification and functional analysis of a novel *IVD* gene variant in this study expanded the *IVD* spectrum. Furthermore, the presence of two or more likely pathogenic or pathogenic variations in an individual with neurodevelopmental phenotypes was not an “anomalous” phenomenon.

## Data Availability

The datasets presented in this study can be found in online repositories. The names of the repository/repositories and accession number(s) can be found below: https://databases.lovd.nl/shared/individuals/00446575.
